# Supplementary material for: Impacts of COVID-19 pandemic on professional identity development of intern nursing students in China: A scoping review
Source: PLoS One. 2022 Oct 13;17(10):e0275387. doi: 10.1371/journal.pone.0275387 (PMC9560130; doi:10.1371/journal.pone.0275387)
Supplement: S2 Table — (DOCX) [file pone.0275387.s002.docx]

| .  S2 Table Quality appraisal of the articles | | | | | | | | | | |  |  |  |  |
| --- | --- | --- | --- | --- | --- | --- | --- | --- | --- | --- | --- | --- | --- | --- |
|  | Q1 | Q2 | Q3 | Q4 | Q5 | Q6 | Q7 | Q8 | Q9 | Q10 | | Q11 | Q12 | T |
| 1. Sun et al.（2021) | Y | Y | N | Y | N | N | Y | Y | Y | N | | N | Y | 7 |
| 1. Ma et al. (2020) | Y | Y | Y | Y | N | N | Y | Y | Y | N | | N | Y | 8 |
| 1. Rao et al. (2021) | Y | Y | Y | Y | N | N | Y | Y | Y | N | | N | Y | 8 |
| 1. Yang et al. (2020) | Y | Y | Y | Y | N | N | Y | Y | Y | N | | N | Y | 8 |
| 1. Yang et al. (2022) | Y | Y | Y | Y | Y | N | Y | Y | Y | N | | N | Y | 9 |
| 1. Zhou et al. (2020) | Y | Y | Y | Y | C | N | Y | Y | Y | N | | N | Y | 8 |
| 1. Hu et al. (2022) | Y | Y | Y | Y | Y | N | Y | Y | Y | N | | N | Y | 9 |
| 1. Luo et al. (2021) | Y | Y | Y | Y | N | N | C | Y | Y | N | | N | Y’ | 7 |
| 1. Tang et al. (2020) | Y | Y | N | Y | N | N | Y | Y | Y | N | | N | Y | 7 |
| 1. Shen et al. (2021) | Y | Y | Y | Y | Y | N | Y | Y | Y | N | | N | Y | 9 |
| 1. Gao et al. (2020) | Y | Y | Y | Y | Y | N | Y | Y | Y | N | | N | Y | 9 |
| 1. Wen et al. (2021) | Y | Y | N | Y | N | N | Y | Y | Y | N | | N | Y | 7 |
| 1. Liu et al. (2020) | Y | Y | Y | N | Y | N | Y | Y | Y | N | | N | Y | 9 |
| 1. Zhang et al. (2021) | Y | Y | Y | Y | N | Y | Y | Y | Y | N | | N | Y | 9 |
| 1. Ruan (2021) | Y | Y | Y | Y | N | Y | Y | Y | Y | N | | Y | Y | 9 |
| 1. Liu et al. (2020) | Y | Y | N | Y | N | N | C | Y | Y | N | | N | Y | 6 |
| 1. Li et al. (2022) | Y | Y | N | Y | Y | N | Y | Y | Y | N | | N | Y | 8 |
| 1. Huang et al. (2021) | Y | Y | N | Y | N | N | C | Y | Y | N | | N | Y | 6 |
| 1. Wang et al. (2021) | Y | Y | Y | Y | N | N | C | Y | Y | N | | N | Y | 7 |
| 1. Nie et al. (2021) | Y | Y | N | Y | Y | N | Y | Y | Y | N | | N | Y | 8 |
| 1. Wang et al. (2020) | Y | Y | N | Y | N | N | C | Y | Y | N | | N | Y | 6 |
| 1. Zhang et al. (2021) | Y | Y | Y | Y | Y | N | C | Y | Y | N | | N | Y | 8 |
| 1. Hao & Jin (2020) | Y | Y | N | Y | N | N | Y | Y | Y | N | | Y | Y | 6 |
| 1. Liu et al. (2021) | Y | Y | N | Y | Y | N | C | Y | Y | N | | N | Y | 7 |
| 1. Tang et al. (2021) | Y | Y | Y | Y | Y | N | Y | Y | Y | N | | N | Y | 9 |
| Summary of categories | Y:25  N:0  C:0 | Y:25  N:0  C:0 | Y:15  N:10  C:0 | Y:24  N:1  C:0 | Y:10  N:14  C:1 | Y:2  N:23  C:0 | Y:18  N:0  C:7 | Y:25  N:0  C:0 | Y:25  N:0  C:0 | Y:0  N:25  C:0 | | Y:2  N:23  C:0 | Y:25  N:0  C:0 |  |
| T | 25 | 25 | 15 | 1 | 10 | 2 | 18 | 25 | 25 | 0 | | 23 | 25 | 194 |

Y:yes, N:no, C: can’t tell, T: total score

Q1: Did the study address a clearly focused question / issue?

Q2: Is the research method (study design) appropriate for answering the research question?

Q3: Is the method of selection of the subjects (employees, teams, divisions, organizations) clearly described?

Q4: Could the way the sample was obtained introduce (selection)bias?

Q5: Was the sample of subjects representative with regard to the population to which the findings will be referred?

Q6: Was the sample size based on pre-study considerations of statistical power?

Q7: Was a satisfactory response rate achieved?

Q8: Are the measurements (questionnaires) likely to be valid and reliable?

Q9: Was the statistical significance assessed?

Q10: Are confidence intervals given for the main results?

Q11: Could there be confounding factors that haven’t been accounted for?

Q12: Can the results be applied to your organization?
