# Supplementary material for: Impacts of COVID-19 pandemic on professional identity development of intern nursing students in China: A scoping review
Source: PLoS One. 2022 Oct 13;17(10):e0275387. doi: 10.1371/journal.pone.0275387 (PMC9560130; doi:10.1371/journal.pone.0275387)
Supplement: S3 Table — (DOCX) [file pone.0275387.s003.docx]

S3 Table. The results of included studies

| Author  (year) | Purpose | Result evaluation | Results |
| --- | --- | --- | --- |
| Sun et al.  (2021) | To investigate the status of professional commitment of the participants and analyze the influencing factors to professional commitment. | The Professional Commitment Scale (4 dimensions, 34 items) was used. | The commitment score was 90.55±16.74, accounting for 66.6% of the total score of 136. The influencing factors to professional commitment included: attitude changes to nursing post-pandemic, professional ideological stability, understanding of professional development, interests in news and research development in nursing (P<0.05). |
| Ma et al.  (2020) | To investigate the psychological status of the participants, their PI, and the influencing factors of PI under the COVID-19 pandemic. | The Professional Identity Questionnaire for Nurse Students (PIQNS) was used. | The PI score was 63.12±12.23, accounting for 74.25% of the total score of 85. The dimension of social persuasion scored the highest and the dimension of profession selection autonomy scored the lowest. About 24% of the interns had acute stress disorders attributed to COVID-19. Preference for nursing had an impact on the PI levels (p=0.00). |
| Rao et al.  (2021) | To investigate the PI of the participants and the influencing factors of PI under the COVID-19 pandemic. | The PIQNS was used. | The PI score was 64.9±11.0, accounting for 76.35% of the total score of 85. The influencing factors to the PI included: attitudes from the society towards nursing, national policy related to nurses, workload, and infection risk to COVID (p<0.05). |
| Yang et al.  (2020) | To investigate the PI of the participants and the influencing factors of PI under the COVID-19 pandemic. | The PIQNS was used. | The PI score was 64.56±10.34, accounting for 75.95% of the total score of 85. The dimension of social persuasion scored the highest while the dimension of retention benefits and turnover risk scored the lowest. The influencing factors to the PI included: gender, social practice, participation in school associations, and willingness to support the frontline professionals (p<0.05). |
| Yang et al.  (2022) | To investigate the PI of the participants and the influencing factors of PI in the COVID-19 pandemic. | The Professional Identity Scale was used (5 dimensions, 30 items). | The PI score among the undergraduate interns was 109.27±13.96, accounting for 72.85% of the total score of 150. The dimension of knowledge and attitudes related to nursing scored the highest while professional support of society scored the lowest. The influencing factors of PI included: retention attempts after graduation, nursing as the first choice of major in college application, family support, received humanistic care during the internship, and tutors’ support during the internship (p<0.05). |
| Zhou et al.  (2020) | To investigate the PI of the participants and the influencing factors of PI during the COVID-19 pandemic. | The PIQNS was used. | The PI score among undergraduate interns was 60.78±9.41, accounting for 71.51% of the total score of 85. The influencing factors of PI included: self-choice of nursing as the major, experience of clinical practice, health status, anxiety, social support (p<0.05). |
| Hu et al.  (2022) | To investigate the PI of the participants and the influencing factors of PI during the COVID-19 pandemic. | The PIQNS was used. | The PI score among the undergraduate interns was 66.78±13.54, accounting for 78.56% of the total score of 85. The influencing factors of PI included: age, nursing major as the first choice, willingness to participate in frontline support, family support, preference for nursing, willingness to engage in clinical work (p<0.05). |
| Luo et al.  (2021) | To investigate the PI of the participants and the influencing factors of PI during the COVID-19 pandemic. | The PIQNS was used. | The PI score among the interns was 68.0, accounting for 80.0% of the total score of 85. The influencing factors of PI included: social activities, experiences of hospital trainee/internship, self-stereotype (p<0.05). |
| Tang et al.  (2020) | To explore the impact of participants’ psychology and coping methods on their PI during the COVID-19 pandemic. | The Professional Identification Scale was used (3 dimensions, 10 items). | The interns from the countryside had higher levels of anxiety than those from the urban areas. The coping styles to anxiety were positively associated with the PI levels (p<0.05). |
| Shen et al.(2021) | To explore the status and the influencing factors of psychological capital of the participants and their PI under the COVID-19 pandemic. | The Psychological Capital Instrument and the Professional Identity Scale (5 dimensions, 30 items) were used. | The psychological capital and its influences were reported. The PI score was 105.39±18.45, accounting for 70.26% of the total score of 150. The influencing factors of PI included: psychological capital, the first choice of nursing as college major, retention intention after graduation, contact with COVID-19 cases in the clinical ward (p<0.05). |
| Gao et al.  (2020) | To identify the association between perceived stress of the participants and their PI under the COVID-19 pandemic. | The Perceived Stress Scale and the Professional Identification Scale (3 dimensions, 10 items) were used. | The score of perceived stress was 44.96±14.52, accounting for 38.76% of the total score of 116. The perceived stress was negatively associated with PI. The PI score was 38.43±6.49, accounting for 76.85% of the total score of 50. The influencing factors of PI included: educational levels, training on knowledge of COVID-19, stress from teaching and nursing staff, stress from peers and daily life, and stress from the clinical environment (p<0.01). |
| Wen et al.(2021) | To compare PI levels of the participants before and after the COVID-19 epidemic. | The PIQNS was used. | While the PI levels of the interns at the post-pandemic time were higher than those at the pre-pandemic time, the difference was not statistically significant.  The factors positively affected the PI included: the Chinese Communist Party membership, being a student cadre, voluntary choice of nursing as a college major, retention in nursing after graduation (p<0.05). |
| Liu et al.  (2020) | To explore the correlation between COVID-related knowledge, attitudes, and practice (KAP) of the participants and their PI. | The COVID-19 KAP scale (3 dimensions and 39 items) and the Professional Identity Scale (One dimension,11 items) were used. | The scores on the KAP scale were positively associated with the PI levels (r=0.45, p<0.01). The PI score was 39.27.±6.18, accounting for 78.54.% of the total score of 50. |
| Zhang et al.(2021) | To investigate the status of the participants’ PI during the COVID-19 outbreak, and to explore the association between PI levels and participants’ attitudes toward public health emergencies. | The PIQNS and the Emergency Attitude Scale for Public Health Emergencies (4 dimensions, 10 items) were used. | The score for public health emergencies was 3.95±0.54, accounting for 79.00% of the total score of 5. The emergency attitudes to public health emergencies were positively associated with PI (r=0.588, P<0.01). The PI score was 64.04±12.02, accounting for 75.34% of the total score of 85. |
| Ruan  (2021) | To investigate the PI of the participants and the influencing factors of PI during the COVID-19 pandemic, which was regarded as a public health emergency. | The PIQNS and the Public Health Emergency scale were used. | The PI score was 58.43±13.83, accounting for 68.74% of the total score of 85. The influencing factors of PI included: admission of COVID-19 patients in the hospital and organization of social activities in school (p<0.05). Some 74% of the interns had psychological deviation disorders in the dimension of fear; 58.8% had a deviation disorder in the dimension of illness suspension. The PI had negatively associated with the five factors in the psychological reaction intensity. |
| Liu et al.  (2020) | To investigate the PI status of the participants and the relationship between job remodeling and PI. | The PIQNS and the Job Remodeling scale were used. | The average score of PI in each of the items was 3.77.±0.57, accounting for 75.40% of the full score of 5. A positive relationship between PI and job remodeling was detected. |
| Li et al.  (2022) | To investigate the PI status of nursing students after the COVID-19 pandemic. and to identify the correlation between the students’ humanistic care ability and their PI. | The Professional Identity Scale (5 dimensions and 30 items) and Caring Ability Scale (3 dimensions and 37 items) were used. | The score of caring ability was 175.93±16.33, accounting for 67.93% of the total score of 259. The PI score was 106.96±14.07, accounting for 71.30% of the total score of 150. There was a positive correlation between caring ability and PI (P<0.05).  The influencing factors of PI included: Nursing as the first choice in college application, and the intention to work as clinical nurse after graduation. |
| Huang et al. (2021) | To investigate the recognition of professional self-concept, analyze the relationship between self-concept, professional commitment and PI, and analyze the influence of intersects in nursing on profession acceptance during the COVID-19 epidemic. | The Professional Commitment Scale (4 dimensions, 34 items), the PIQNS, and the Professional Self-concept of Nursing Interns (5 dimensions, 30 items) were used. | The total score of professional commitment score was(97.44±12.94, accounting for 71.65% of the total score of 136. The PI score was 64.49±11.59, accounting for 75.87% of the total score of 85. The score of professional self-concept was 88.10±13.56, accounting for 73.42% of the total score of 120. The professional commitment, the PI and the interests in nursing were positively related to the professional self-concept of nursing(P＜0.05). |
| Wang et al.  (2021) | To explore the PI levels among the participants, the influencing factors of PI, the participants’ status of psychological resilience and to identify the correlation between the PI and psychological resilience during the COVID-19 epidemic. | The Connor-Davidson Resilience Scale and the PIQNS were used. | The PI score was 60.84± 11.27, accounting for 71.58% of the total score of 85. The resilience score was 25.17± 6.54, accounting for 62.93% of the total score of 40. A positive relationship between the PI and the resilience was revealed(P<0.05). The psychological resilience, preference for nursing major, future employment intention, grade, and gender could explain the 52. 1% of the variation in nursing students’ PI. |
| Nie et al.  (2021) | To investigate participants’ PI, intention to leave nursing, and the perception of clinical nursing work during the COVID-19 pandemic and to explore the factors influencing the PI. | The PIQNS was used. | The PI score was 3.95 for each item, accounting for 79.00% of the total score of 5. The influencing factors of PI included: intention to stay in nursing, spending time on COVID-19 events, having seen COVID cases, COVID-19 related knowledge, perceived importance of prevention and control measures against COVID, inspiration attributed to the COVID-19 pandemic. |
| Wang et al.  (2020) | To evaluate the participants’ PI, self-efficacy, and acute stress response during the COVID-19 outbreak, and to investigate the effect of the acute stress response on PI and self-efficacy. | The PIQNS, the Generalized Self-efficacy Scale (10 elements), and the Stanford Acute Stress Response Scale Questionnaire (30 self-report measures) were used. | The PI score was 3.80±0.67, accounting for 76% of the total score of 5. The score of self-efficacy was 2.60 ± 0.59, accounting for 65% of the total score of 4. The score of acute stress response was 2.22±0.99, accounting for 44.40% of the total score of 5. The self-efficacy had a positive effect on the PI (p<0.001) while acute stress response had a negative effect on the PI and self-efficacy, respectively (p<0.001). |
| Zhang et al.  (2021) | To describe the sense of PI among the participants during the COVID-19 outbreak and to explore the relationship between the psychological resilience and the sense of PI. | The PIQNS and the Connor-Davidson Resilience scale were used (10 items). | The PI score was 62.02 ± 12.02, accounting for 72.96% of the total score of 85. The score of the psychological resilience was 35.41 ± 8.29, accounting for 70.82% of the total score of 50. Psychological resilience was the strongest contributor to the PI (β = 0.371, P < 0.001). About 86% of the participants attributed the elevated nursing image to the COVID-19 pandemic. The positive influencing factors of PI included: better psychological resilience, in the second to the fourth school year, studying in the place once experienced the COVID pandemic, ranked nursing as their first choice of the college application, studying nursing by own interest, parents being not medical workers, lower monthly expenditures, and regarding COVID-19 as a positive influence on the nursing image (P < 0.05). |
| Hao et al.  (2020) | To investigate the PI of the participants and the influencing factors to the PI during the COVID-19 pandemic. | The PIQNS was used. | The PI score was 61.37 ± 13.03, accounting for 72.2% of the total score of 85. The influencing factors of PI included: Gender, nationality, education level, and whether they had been a volunteer during the pandemic (P<0.05). The participants who were proud to be in nursing scored the highest of the PI. |
| Liu et al.  (2021) | To understand the participants’ professional attitudes, their PI, and their employment intention under the COVID-19 pandemic, and to analyze the factors influencing their employment intention. | A self-designed questionnaire containing four parts: basic information, professional attitude, employment intention, and open questions . | Only 0.73% of the nursing students actively chose to work in the infectious disease unit in the background of the COVID-19 pandemic. The influencing factors to professional attitudes included: gender, grade, internship experience, and connections to someone involved in the rescue work of COVID-19 cases (P<0.05). The influencing factors to the PI included: grade, family monthly income, reasons for choosing nursing as a college major, and clinical practice experience(P<0.05). |
| Tang et al.  (2021) | To investigate the factors associated with the participants’ PI during the epidemic of COVID-19. | The PIQNS was used. | The PI score was 59.49 ± 12.41, accounting for 69.99% of the total score of 85. The influencing factors of PI included: gender, residential area, major, the impact of the epidemic on intention to work after graduation, reasons for choosing nursing as a college major, and the PI score at the beginning of nursing education. |
