# Supplementary material for: Impacts of COVID-19 pandemic on professional identity development of intern nursing students in China: A scoping review
Source: PLoS One. 2022 Oct 13;17(10):e0275387. doi: 10.1371/journal.pone.0275387 (PMC9560130; doi:10.1371/journal.pone.0275387)
Supplement: Attachment — Submitted filename: Response to reviewers.docx [file pone.0275387.s004.docx]

Response to reviewers

We express sincere thanks to the reviewer who must have invested much time in our paper and provided valuable suggestions on the way to improve the quality of the paper.

The reviewer suggested a major revision and we have tried our best to address the reviewer’s concerns.

Here are our responses to Reviewer1’s comments.

| **Reviewer1’s comments** | **Authors’ responses** |
| --- | --- |
| This is an interesting paper on the potential impact of COVID-19 on the professional identity of nursing students in China. Much discussion has been given to the role of the pandemic on the essential healthcare workforce, potentially contributing to burnout, anxiety, and perceptions of a lack of support from the general public. Overall the concept of the study is novel and contributes to the literature. The methods of the scoping review are sound and the approach is justified. The introduction and background is largely unsupported and many statements are made without citation or evidentiary support. It is confusing why there are two sections when a more traditional introduction section of a scientific paper would suffice. Regardless as to whether it is a unified or separate approach, however, there are too many statements made without citation. The results needs substantial revision as the presented findings do not fully reflect the objectives of the study. | Many thanks for the positive comments on our paper.  We have tried our best to address the weaknesses raised by the reviewer. |
| Introduction |  |
| Replace “has posed” to “pose” to avoid passive voice | We have modified the sentence.  Page 5. Lines 107-108. |
| - Line 107, replace “past two years” with the actual years, because if this paper is published and someone reads it 5 years from now, it is now inappropriate. | We have replaced the words “past two years” with the year 2019.  Page 5. Line 108. |
| - Line 108, “It may also affect the …intern nursing students…” Is this a declaratory statement? It does not have a citation. If it is something you are trying to prove, then that is acceptable but should be listed in objectives instead. If it is just a declaratory statement, it needs citation or needs removal | We have modified the sentence and added citations.  Page 5. Lines 108-110. |
| - Line 108, similarly without citation and superfluous | We have added citations.  Page 5. Line 110 |
| - Citation needed for sentence ending on line 112 | Citations were provided.  Page 5. Lines 115 |
| - Statements from line 113 – 117 reference studies but these are not cited. | We have modified the sentences. |
| - Please define what an intern nursing student is. | The definition was provided.  Page 5. Lines 118-120 |
| - Related to above, be clear that if this study uses international citations, whether the intern nursing student definition is context-specific | We have made it clear that the definition is under China-context. |
| Background |  |
| - Why is there a background section and this not part of the introduction? Much of this seems to share similarity with the Introduction. If this is an editorial requirement of PLOS One then please ignore my comment. If it is not, these two sections (intro and Background) should be combined, trimmed, and revised for ease of reading. | We have combined the two sections and made some revisions after the combination to avoid repetitions.  Page 5. Lines 104-117 |
| - Page 7, line 134: “alternations” is a confusing word choice | We have changed the words.  Page 6. Line 133 |
| - Page 7, lines 137-142. This is an uncited, un-referenced paragraph. The “up to down” governance in China may be familiar to the author but it will not be to any reader outside of China. Additionally, there is no evidentiary support given for these statements. Especially lines 141-142. | We have modified this paragraph and added citations.  Page 6. Lines 137-142 |
| - Line 143-145, again, no citation for a statement that needs it - Line 145-156, studies are mentioned but not referenced | We have added citations.  Page 6. Line 146 |
| Methods |  |
| - The first research question on line 162 is not clear. “How was the PI status?” is there a word missing? | We have revised the wording.  Page 7. Lines 161-162 |
| - Please include the entire PubMed/MEDLINE search strategy, not broken into boxes as shown in Figure 1. The search must be able to be replicated exactly. | We deleted Fig 1 and described the search strategy in detail.  Page 8. Lines 170-174 |
| - Lines 181-190 need a rewrite for clarity and cohesion | This section has been rewritten.  Pages 8. Lines 183-189 |
| Results |  |
| - As presented, Table 1 is illegible. Half of the first column is missing, as is the last column | We have re-formatted Table 1. |
| - Table 2 needs some kind of massive revision. As presented, Table 2 is 19 pages long. The authors should consider a shorter, summary Table 2 and putting much of the rest of the information in the Appendix | As suggested, we have established a shorter table with basic information about the included studies and another longer table with detailed information on the study findings. The longer table is an appendix.. |
| - Related to the above comment about Table 2, the Results section needs revision. With a scoping review, much information is generated. It is unclear why the section on the instrumentation is needed, as it does not seem to provide much in regards to the selected research questions. | We modified Table 2 and decided to keep some information on instruments in the appendix table because the instruments were evaluating tools of students’ PI. |
| - Much like the issue with instrumentation, if the paper is focused on nursing PI in response to COVID-19, then the entire sections on personal and social factors is superfluous. It is not relevant to discuss gender, family, or interpersonal impacts on PI if these are completely unrelated to COVID-19, which they seem to be. Based off these large pieces of this review, it is recommended that the authors substantially revise the Results section. Table 1 and 2 need to be revised. As well, the authors have presented some interesting findings on the nurse PI/COVID dynamic, and this can (and should) be expanded further. By removing the unnecessary findings on non-COVID PI findings, the authors can then spend much more time synthesizing and summarizing the study findings on PI/COVID, which is the point of the paper. | One of the two research questions of the review study is “What factors affected the PI of intern nursing students?” . We decided to report the findings on personal and social influences.  On the other hand, we agree with the reviewer’s point that these findings do not have much relevance to COVID-related impacts. We substantially shortened the reports of the findings.  Pages 17-18. Lines 269-281 |
| - The results, as presented, do not match the theoretical framework for the qualitative approach described by the authors in Methods. | We have modified the result. |
| Discussion |  |
| - Line 359-363 are unsubstantiated | We have added citations.  Page 18. Lines 290-291 |
| - The discussion on the instrumentation largely reflects the confusion and mis-placement of the instrumentation of Results. The study is on the impact of COVID-19 on nurse professional identity. It seems unsupported by the results to connect changes in this PI based on instrumentation, when the studies themselves were not reflective of COVID-19 | We agree with the reviewer's point. However, we did not discuss the instruments per se. Instead, we discussed the findings evaluated by the instruments. |
| - Line 436-440, “wishful thinking” is not a scientific statement | We have revised the words.  Page 24. Line 409 |
